# Lipidomics and biochemical profiling of adult Yili horses in a 26 km endurance race: exploring metabolic adaptations

**DOI:** 10.3389/fvets.2025.1597739

**Published:** 2025-04-22

**Authors:** Xiaokang Chang, Zihan Zhang, Xinkui Yao, Jun Meng, Wanlu Ren, Yaqi Zeng

**Affiliations:** ^1^College of Animal Science, Xinjiang Agricultural University, Urumqi, China; ^2^Xinjiang Key Laboratory of Equine Breeding and Exercise Physiology, Urumqi, China

**Keywords:** Yili horse, lipid metabolomics, blood biochemistry, endurance racing, regulatory mechanisms

## Abstract

The equine lipid metabolism is activated during and after endurance exercise to provide energy in response to the metabolic and physiological changes in the body caused by prolonged exercise; however, the specific regulatory mechanisms remain controversial and identifying differential lipid metabolites associated with equine endurance is essential to elucidate these regulatory mechanisms. In this study, blood samples for lipid metabolomic analysis and biochemical indices were collected before and after a 26 km race from 12 Yili horses with different endurance performance. The biochemical results showed that: the albumin (ALB) level was significantly higher in the general group than in the excellent group before the competition, but significantly lower in the ordinary group after the competition (*p* < 0.05); the pre-competition alanine aminotransferase (ALT) in the excellent group was significantly higher than that of the general group (*p* < 0.05); and the urea nitrogen (BUN) in the general group was significantly higher than that of the excellent group after the competition (*p* < 0.05). The lipid metabolism results showed that a total of 1,537 lipid differential metabolites were obtained, mainly enriched in the pathways of fatty acid biosynthesis, cortisol synthesis and secretion, bile secretion, aldosterone regulation of sodium reabsorption, biotin metabolism, steroid hormone biosynthesis, and neuroactive ligand-receptor interactions. Metabolomics and biochemical correlation analyses screened PC (18:3/18:4) and PI (18:1/18:2) as potential biomarkers to identify endurance performance in Yili horses. The results of this study provide a solid foundation for improving equine racing performance and for the selection and breeding of endurance horses by providing a comprehensive reference on the mechanisms of lipid metabolism in equine endurance.

## 1 Introduction

Equine endurance racing places high demands on the physiological adaptations of the horse, and endurance exercise in horses results in increased cardiac output and fluid loss, which can lead to severe imbalances in homeostasis (1). Metabolomics is an important tool for understanding the metabolic response of athletes to prolonged strenuous exercise (2–5). Some studies have found (6), Twenty-three metabolites were altered in horses after moderate-intensity exercise, while 54 differential metabolites associated with pathways such as amino acid, fatty acid, nucleic acid, and vitamin metabolism were identified after high-intensity exercise. Klein (7) found that 142 metabolites changed significantly after 3 h of treadmill exercise in horses. Therefore, an in-depth study of the molecular regulatory mechanisms and physiological states of endurance racehorses under sustained exercise can provide a theoretical basis for enhancing the horse’s race performance and optimizing training programs.

Lipidomics is considered a subfield of metabolomics, focusing on the study of the composition, structure, and function of all lipid molecules in an organism and their changes during physiological and pathological processes (8–10). Lipid metabolism is a central process of energy storage, cellular structure and signaling in organisms and is extremely important for aerobic and anaerobic exercise (11), causing strong changes in lipid metabolites during and after endurance exercise (12, 13). Previously Nolazco (14) used an untargeted lipidomic approach to compare changes in the plasma lipidome of Thoroughbreds before and after ultramaximal exercise and found that 13 lipids were altered after exercise, including seven fatty acids (FAs) and three phosphatidylcholines (PCs), one lysophosphatidylcholine (LPC) and two sphingomyelins (SMs). However, this study had a small sample size (four Thoroughbreds) and the results were limited to the finding that high-intensity exercise induces changes in the plasma lipidome of racehorses, and there was no deeper dive into the biomarkers associated with endurance performance. Since there is some variability in the athletic performance of different horses breed, which makes their race performance and body metabolism different, further studies on how lipids are supplied as energy in horses are needed to better elucidate the pathways related to lipid metabolism.

The Yili horse is the only riding horse breed independently bred in China. Through long-term complex multi-breed crossbreeding and selection under unchanged grazing conditions and continuous improvement, it has the characteristics of strong resistance to adversity and good endurance performance, and has become one of the main racing breeds in Xinjiang, China (15, 16). In this study, we investigated the differences in plasma lipid metabolites before and after 26 km endurance races of different endurance horses through lipidomics, analyzed the energy supply pathways of endurance horses by combining blood biochemical indexes, and gained an in-depth understanding of the ways in which energy is produced and utilized by lipid metabolism in long-distance exercise, which provides scientific evidence for the in-depth understanding of the physiological mechanisms of the endurance horse, the improvement of endurance performance, and the refinement of selection, breeding and management strategies for endurance horses.

## 2 Materials and methods

### 2.1 Animal and sample collection

In this study, horses participating in the 26 km traditional endurance race for Yili horses were selected as research subjects. The rules of the competition are strictly enforced in accordance with the “Rules of Racing of the Chinese National Horse Race Traditional Endurance Race”. All participating horses have a Yili Horse Registration Passport issued by the Xinjiang Horse Industry Association, aged between 5 and 10 years, underwent a pre-race veterinary examination to confirm their good health, with no signs of lameness, metabolic disorders, or fatigue, and after the race the horses that finished in the rankings were tested for prohibited substances. The racecourse consisted of grass terrain for the first 20 kilometers and sandy terrain for the final 6 kilometers. The ranking was determined based on the total time taken to complete the 26-kilometer race without stopping, and the results were recorded simultaneously.

Twelve adult male horses that completed the race and finished in the top six (excellent group) and bottom six (ordinary group), respectively, were selected as experimental animals from 207 participating horses, and blood was collected from the jugular vein of the horses the day before the race and after the race (when the heart rate recovered to 64 beats/min and below) (17), with two tubes (sodium heparin and vacutainer) of 5 mL each at a time. All blood samples were prepped immediately after collection and plasma was extracted by centrifugation through sodium heparin centrifuge tubes at 3500 r/min for 15 min and then stored in liquid nitrogen for lipid metabolite extraction (18). Another 5 mL of empty blood samples were allowed to stand for 10 min and then centrifuged at 3500 r/min for 15 min to extract serum, which was stored in a − 20°C refrigerator for biochemical analysis (19).

### 2.2 Measurement of biochemical indicators

Total protein (TP), albumin (ALB), total cholesterol (TC), urea nitrogen (BUN), uric acid (UA), creatinine (CREA), and lactic acid (LAC) concentrations were determined using a spectrophotometer and an enzyme marker, as well as the activities of alanine aminotransferase (ALT), alanine oxaloacetyltransferase (AST), creatine kinase (CK) and lactate dehydrogenase (LDH) activities. The manufacturers and parameters of the instruments are listed in Table 1.

**Table 1:** Biochemical indexes determination kits and test instruments.

Serial number	Biochemical index	Kit	Laboratory Instruments	Manufacturer
1	Total Protein	A045-1-1 Total Protein Assay Kit	752N UV–Vis Spectrophotometer	Shanghai Yidian Analytical Instrument Co.
2	Lactic acid	A019-2-1 Lactic acid assay kit		
3	Creatine kinase	A032-1-1 Creatine kinase assay kit		
4	Urea Nitrogen	C013-1-1 Urea Nitrogen Assay Kit		
5	Albumin	A028-2-1 Albumin Assay Kit	HBS-1096A Enzyme Labeling Analyzer	Nanjing DeTie Experimental Equipment Co.
6	Lactate dehydrogenase	A020-2-1 Lactate dehydrogenase assay kit		
7	Alanine aminotransferase	C009-2-1 Alanine aminotransferase assay kit		
8	Aspartate aminotransferase	C010-2-1 Aspartate aminotransferase assay kit		
9	Creatinine	C011-2-1 Creatinine Assay Kit		
10	Uric acid	C012-2-1 Uric acid test kit		
11	Total Cholesterol	A111-1-1 Total Cholesterol Assay Kit		

### 2.3 Biochemical data analysis

The biochemical data obtained were organized using Microsoft Excel, and the data were statistically tested based on SPSS 25.0 software, and the results of the biochemical tests before and after the race and the horses’ performance in the excellent and ordinary groups were subjected to the Independent-Samples T Test (IST), and the statistical results were expressed as the mean ± standard error (Mean ± SE), with *p* < 0.01 being highly significant and *p* < 0.05 being significant (19).

### 2.4 Metabolite extraction

100 μL of liquid sample was added to a glass centrifuge tube with a PTFE-lined cap, 0.75 mL of pre-cooled methanol was added, and vortexed and shaken. Add 2.5 mL of pre-cooled methyl tert-butyl ether and incubate on a shaker at room temperature for 1 h. Add 0.625 mL of mass spectrometry-grade water and mix well to stratify the organic phase, and incubate at room temperature for 10 min before centrifuging for 10 min at 1,000 r using a Thermo ST16R cryogenic centrifuge. Collect the upper organic phase (MTBE) and add 1 mL of mixed solvent (methyl tert-butyl ether/methanol/water) (10:10:10) to the lower (water and methanol) layer. The upper organic phase was collected by adding 1 mL of mixed solvent [methyl tert-butyl ether/methanol/water (10:3:2.5, v/v/v)] to the lower layer (water and methanol) and extracted again. The two collected organic phases were concentrated by nitrogen blowing using a Reacti-Therm nitrogen blower.

Resolutions were performed with 100 μL of isopropanol. The samples were analyzed by LC–MS/MS system using Q ExactiveTM HF mass spectrometer, Thermo VanquishTM UHPLC chromatograph by liquid-mass spectrometry (LC–MS) technique (20). An equal amount of supernatant was taken from each processed sample and mixed as QC sample. The lipid metabolomics sequencing was performed by Beijing Novogene Bioinformatics Technology Co., Ltd.

### 2.5 Metabolite data processing and statistical analysis

The raw downlink data (raw) files were imported into Compound Discoverer 3.1 library search software for simple screening of retention time, mass-to-charge ratio and other parameters, and then peaks were aligned according to retention time deviation of 0.2 min and mass deviation of 5 ppm for different samples, followed by peak extraction according to the settings of mass deviation of 5 ppm, signal intensity deviation of 30%, and signal-to-noise ratio of 3, Minimum signal intensity 100,000, summed ions and other information for peak extraction, as well as quantification of peak area, and then integration of the target ions, followed by molecular formula prediction by molecular ion peaks and fragmentation ions and comparison with the Lipidmaps and Lipidblast databases, removal of background ions by blank samples, and normalization of the quantitative results to lipid data The results were characterized and quantified (21).

The data were transformed using the software metaX and then subjected to Partial Least Squares Discrimination Analysis (PLS-DA) to obtain the VIP value for each metabolite. Statistical significance (*p*-value) of each metabolite between the two groups was calculated based on the t-test, and the Fold Change (FC value) of the metabolite difference between the two groups was calculated. The Variable Importance for the Projection (VIP) obtained from the PLS-DA model was used to measure the strength of the influence of the expression pattern of each metabolite on the categorical discrimination of the samples in each group and the explanatory ability to mine biologically significant differential lipid metabolites, and the criteria for the screening were VIP > 1, *p* < 0.05 and FC > 1.2 or FC < 0.5, respectively. 1.2 or FC < 0.833. Pathways of differential lipid metabolites were identified using KEGG database analysis.

### 2.6 Correlation analysis

The obtained biochemical data and differential lipid compounds were subjected to correlation analysis by the correlation analysis tool of the Maiwei Cloud Platform,^1^ which calculates the correlation between different substances based on the quantitative data of the substances of interest, and then draws a correlation heat map to visualize the magnitude of the correlation between the substances (22).

## 3 Results

### 3.1 Analysis of differences in race results

The results of the analysis of differences in race performance of different endurance performance Yili horses (Table 2) showed that the time taken by the excellent group was significantly lower than that of the ordinary group (*p* < 0.05).

**Table 2:** Results for Yili horses with different stamina.

Grades	Excellent group	Ordinary group	*P*-value
Time(s)	2534.00 ± 9.88^b^	3667.17 ± 119.55^a^	0.019

Peer shoulder labeling with different lowercase letters indicates significant differences (*P* < 0.05), different capital letters indicates highly significant differences (*P* < 0.01), and no labeling indicates non-significant differences (*P* > 0.05), the same below.

### 3.2 Blood biochemistry analysis

#### 3.2.1 Analysis of the differences in pre-game biochemical indexes between the excellent group and the ordinary group

The analysis of the differences in blood biochemical indexes before the 26 km endurance race between the excellent group and the ordinary group (Table 3) showed that the TP, LAC, LDH, ALT and CREA contents in the ordinary group were higher than those in the excellent group but the difference was not significant, and the TC and CK contents in the excellent group were higher than those in the ordinary group but the difference was not significant. The levels of ALB and UA were significantly higher in the average group than in the excellent group before the race, and the levels of ALT were significantly higher in the excellent group than in the average group.

**Table 3:** Differential analysis of pre-race blood biochemical indexes between the excellent group and the ordinary group of 26 km endurance race.

Blood biochemical index	Excellent group	Ordinary group	*p*-value
TP (g/L)	70.66 ± 2.53	73.83 ± 2.12	0.681
ALB (g/L)	19.88 ± 0.87^b^	23.10 ± 0.65^a^	0.014
TC (mmol/L)	1.92 ± 0.11	1.90 ± 0.07	0.545
ALT (U/L)	6.63 ± 0.84^a^	3.68 ± 0.50^b^	0.026
AST (U/L)	79.11 ± 9.64	91.40 ± 13.86	0.240
CK (U/ml)	0.22 ± 0.04	0.17 ± 0.01	0.052
LDH (U/L)	2286.38 ± 160.74	2446.01 ± 136.93	0.688
BUN (mmol/L)	4.73 ± 0.15^b^	6.31 ± 0.33^a^	0.025
UA (μmol/L)	197.43 ± 34.96	165.74 ± 17.38	0.056
CREA (μmol/L)	85.42 ± 7.67	86.56 ± 4.66	0.057
LAC (mmol/L)	2.07 ± 0.14	2.28 ± 0.13	0.824

Peer shoulder labeling with different lowercase letters indicates significant differences (*P* < 0.05), different capital letters indicates highly significant differences (*P* < 0.01), and no labeling indicates non-significant differences (*P* > 0.05).

#### 3.2.2 Analysis of the differences in biochemical indexes between the excellent group and the ordinary group after the race

After 26 km endurance exercise, ALB in blood was significantly higher in the excellent group than in the normal group. Also TP, LAC, ALT, CREA, uric acid and TC showed higher levels in the excellent group, but the difference was not significant. The values of LDH, CK and AST were higher in the ordinary group than in the excellent group, but there was no statistical difference (Table 4).

**Table 4:** Differential analysis of post-event blood biochemical indices of the 26 km endurance race between the excellent group and the normal group.

Blood biochemical index	Excellent group	Ordinary group	*P*-value
TP (g/L)	89.48 ± 2.29	79.27 ± 1.76	0.868
ALB (g/L)	26.51 ± 1.33^a^	23.49 ± 0.66^b^	0.047
TC (mmol/L)	2.73 ± 0.16	2.18 ± 0.11	0.336
ALT (U/L)	7.44 ± 0.76	5.66 ± 0.94	0.381
AST (U/L)	134.37 ± 16.82	141.79 ± 11.82	0.428
CK (U/ml)	0.33 ± 0.03	0.38 ± 0.44	0.521
LDH (U/L)	3568.08 ± 191.42	4056.33 ± 164.73	0.980
BUN (mmol/L)	6.07 ± 0.14	8.84 ± 0.29	0.078
UA (μmol/L)	409.71 ± 61.60	277.80 ± 24.57	0.059
CREA (μmol/L)	149.76 ± 10.27	116.49 ± 7.70	0.501
LAC (mmol/L)	4.63 ± 0.11	2.88 ± 0.10	0.533

Peer shoulder labeling with different lowercase letters indicates significant differences (*P* < 0.05), different capital letters indicates highly significant differences (*P* < 0.01), and no labeling indicates non-significant differences (*P* > 0.05).

### 3.3 Lipid metabolomics analysis

#### 3.3.1 Quality control of data

**Figure 1:**
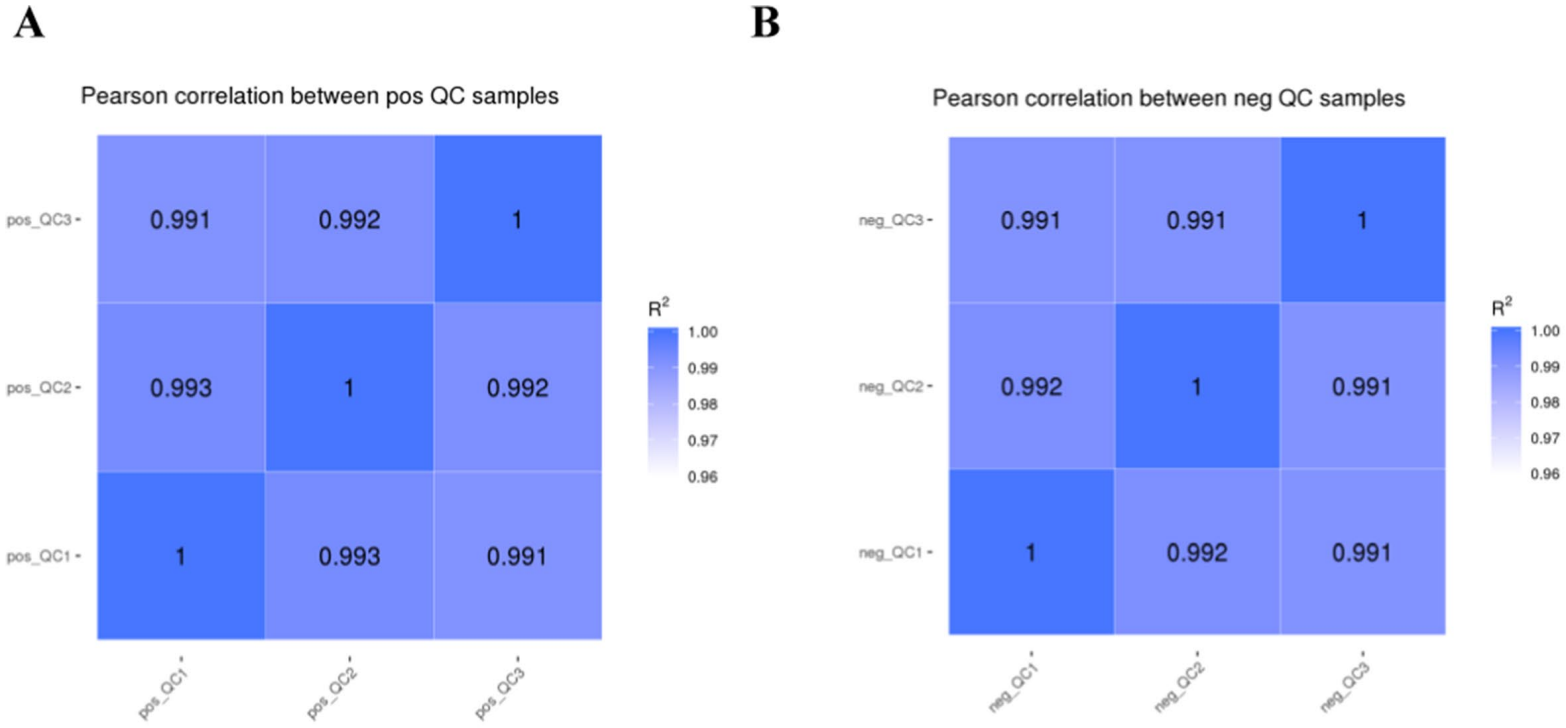
Correlation analysis of QC samples.

The higher the QC sample correlation (R^2^ closer to 1) indicates the better stability of the detection process and the higher quality of the data. The QC sample correlation is shown in Figure 1, and the R^2^ values of this study are all higher than 0.99, which indicates better experimental stability.

#### 3.3.2 Plasma metabolite analysis

**Figure 2:**
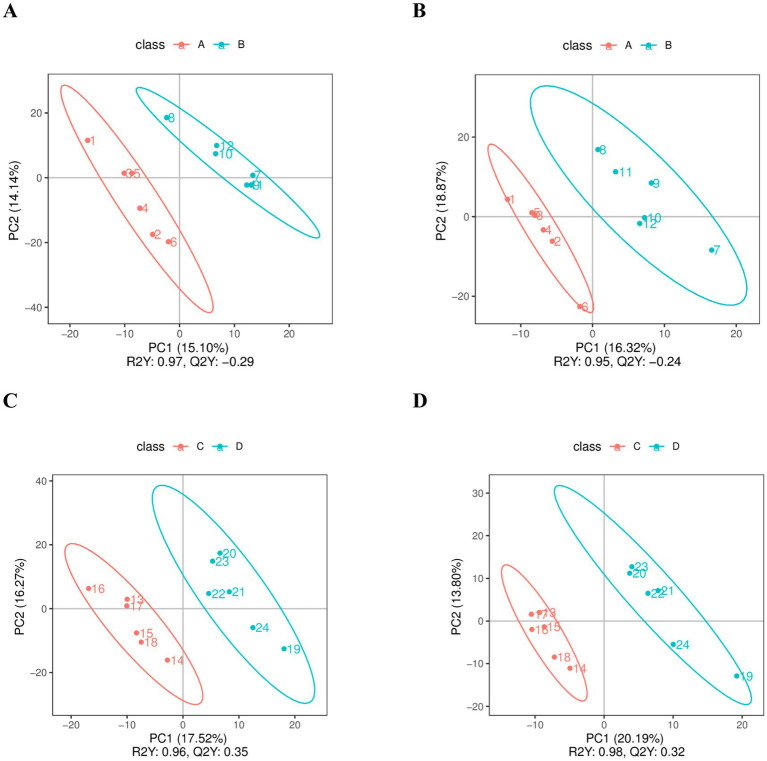
PLS-DA score chart. **(A,C)** are the PLS-DA score charts comparing the pre-game and post-game scores of the excellent and normal groups in the positive mode. **(B,D)** are the PLS-DA score charts comparing the pre-game and post-game scores of the excellent and normal groups in the negative mode.

When comparing Yili horses with different endurance performances, the pre-race Excellent Group is named as Group A, the pre-race Ordinary Group is named as Group B before the race, the post-race Excellent Group is named as Group C, and the post-race Ordinary Group is named as Group D (the same below), and the PLS-DA model was established for each comparative group (Figure 2), and the model evaluation parameters (R^2^, Q^2^) were obtained after seven cycles of interactive validation (7-fold cross-validation), and the model evaluation parameters (R^2^, Q^2^) were obtained if R^2^ and Q^2^ are closer to 1, which indicates that the model is more stable and reliable. In this study, the R^2^ values of the excellent group and the ordinary group were greater than 0.95 in both pre-race and post-race, indicating that the model was more stable.

**Figure 3:**
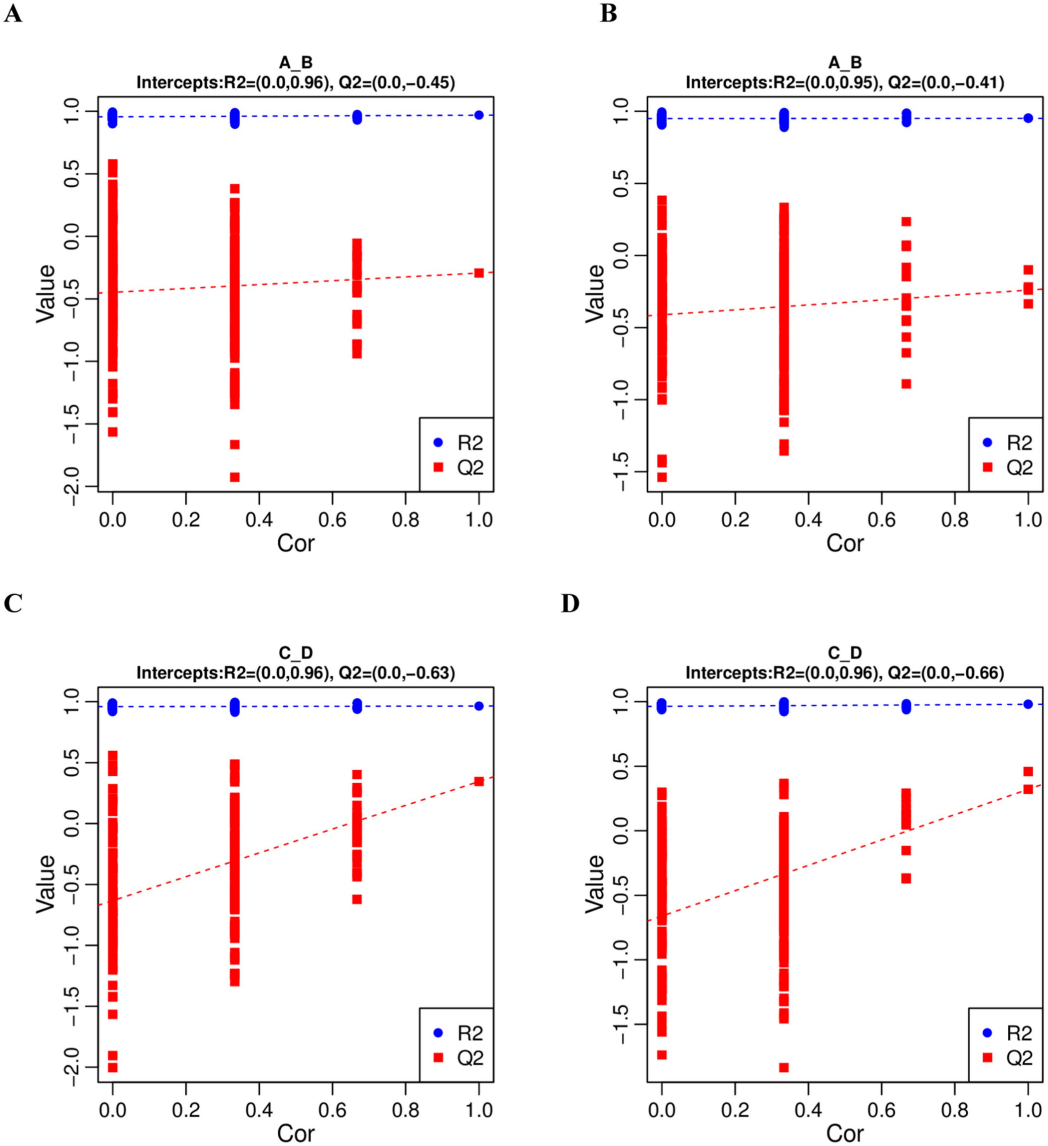
PLS-DA sequential validation map. **(A,C)** represents the PLS-DA sorting validation charts comparing the pre-game and post-game of the excellent and normal groups in the positive mode. **(B,D)** represents the PLS-DA sorting validation charts comparing the pre-game and post-game of the excellent and normal groups in the negative mode.

In order to judge the quality of the model, we sorted the model to verify whether the model was “overfitted”. When the R^2^ data is greater than the Q^2^ data and the intercept of the Q^2^ regression line with the Y-axis is less than 0, it indicates that the model is not “overfitted”. In this study, R^2^ > Q^2^ and the intercept between the Q^2^ regression line and the Y-axis is less than 0, which indicates that the model is not overfitted, and can describe the samples better, which can be used as a prerequisite for finding biomarker clusters (Figure 3).

**Figure 4:**
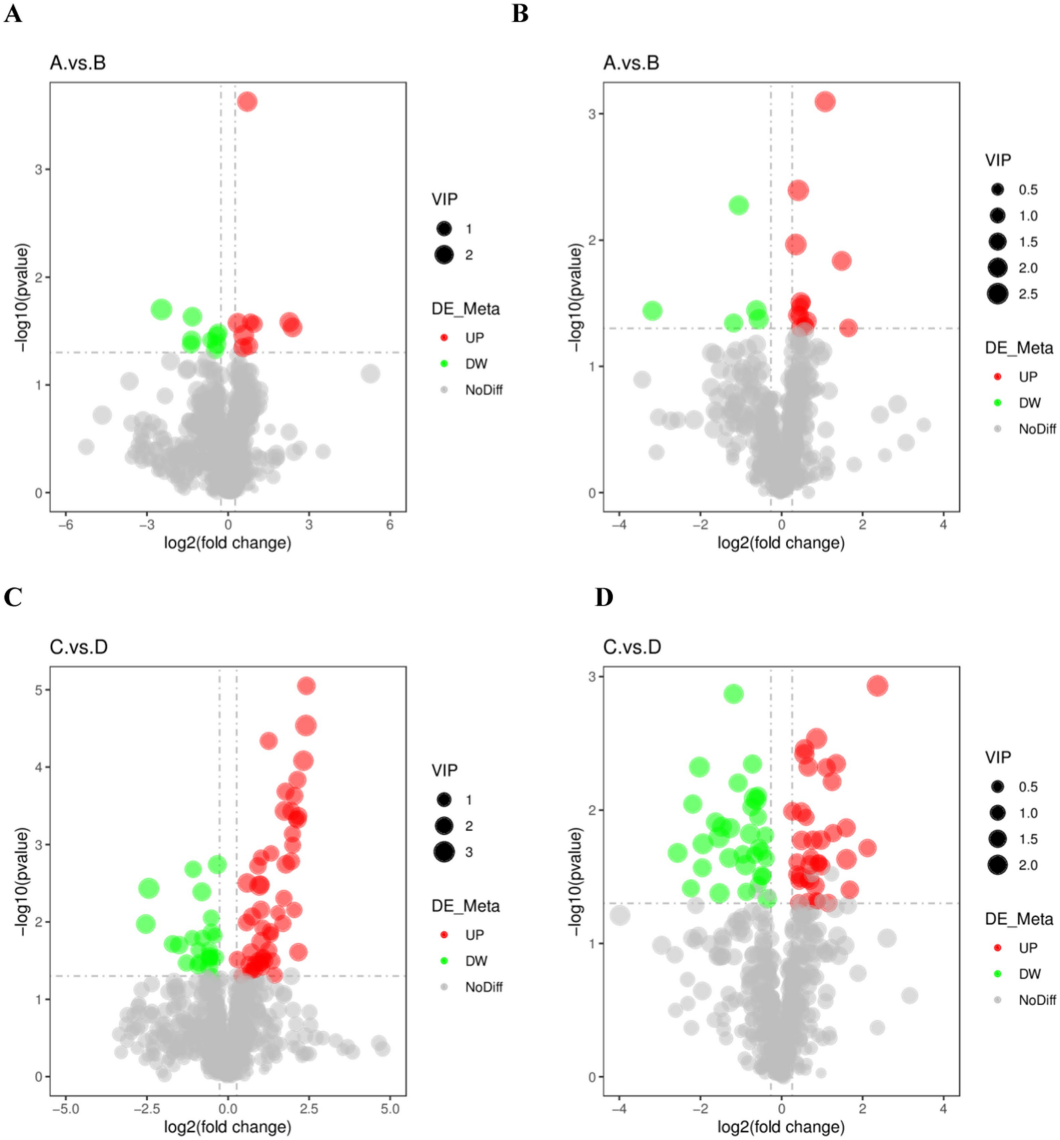
Differential metabolic volcano plot for the excellent group vs. ordinary group. **(A,C)** are the volcano charts in positive mode comparing the pre-game and post-game of the excellent and normal groups. **(B,D)** are the volcano charts in negative mode comparing the pre-game and post-game of the excellent and normal groups.

Metabolites with VIP > 1, FC > 1.2 or FC < 0.833 with *p* < 0.05 in PLS-DA model were selected as differential lipid metabolites. Volcano plots were used to show the overall distribution of differential lipid metabolites for each subgroup, with red dots indicating significantly up-regulated metabolites, green dots indicating significantly down-regulated metabolites, and grey representing metabolites with no difference (Figure 4).

**Figure 5:**
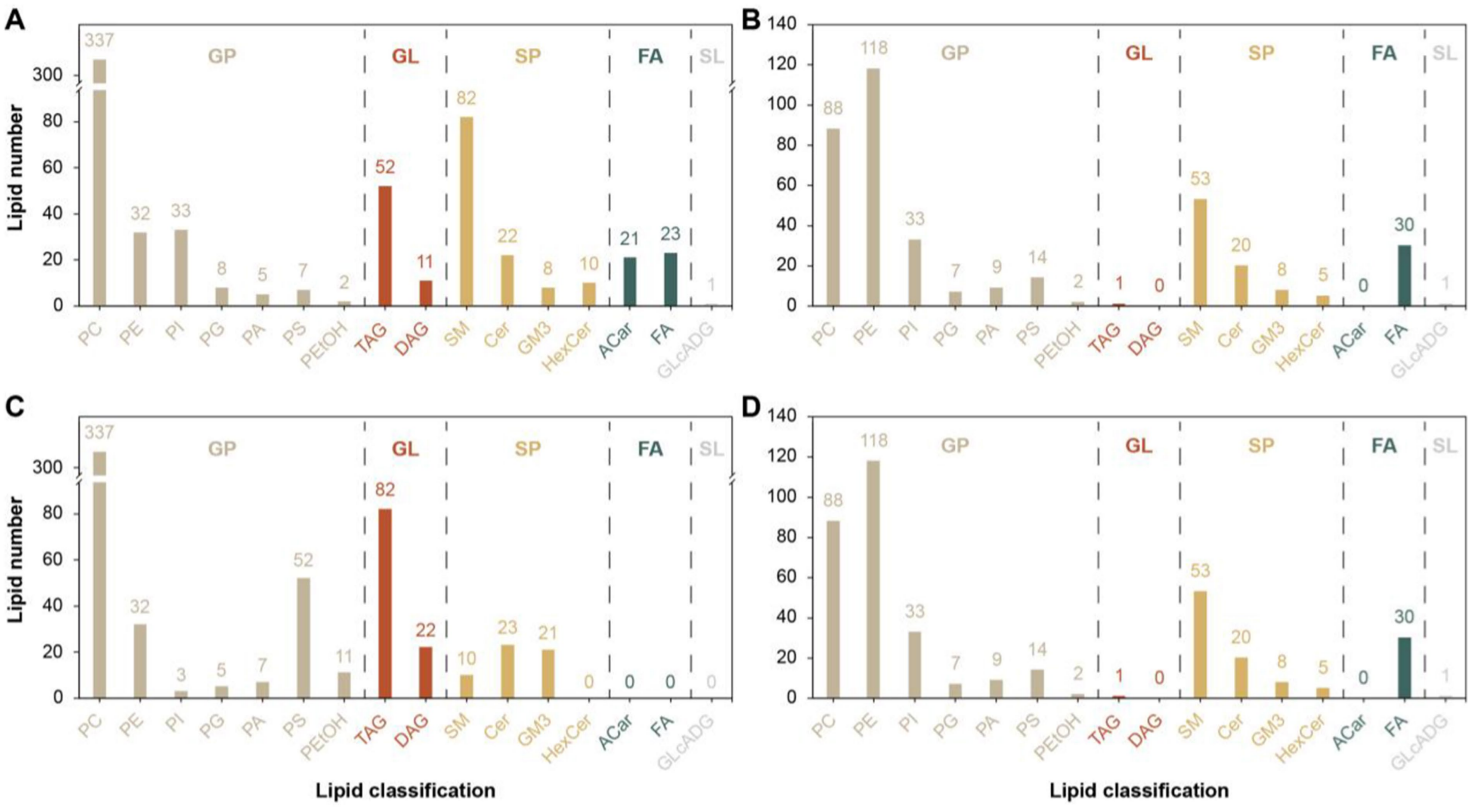
Differential lipid metabolite classification map. **(A,C)** denotes the difference metabolite classification plots in the positive mode for the pre-game and post-game comparisons between the excellent and average groups. **(B,D)** denotes the difference metabolite classification plots in the negative mode for the pre-game and post-game comparisons between the excellent and average groups.

A total of 16 different lipid metabolites were screened in the positive and negative modes before and after the 26 km endurance race of adult Yili horses (Figure 5), including 7 glycerophospholipids (GP), 4 sphingolipids (SP), 2 glycerol esters (GL), 2 fatty acids (FA) and 1 glycolipid (SL). A total of 1,537 differential lipid metabolites were screened before and after the pre-game and post-game in the excellent vs. ordinary group, of which 915 differential metabolites were screened in the positive ion mode and 622 differential metabolites were screened in the negative ion mode.

**Figure 6:**
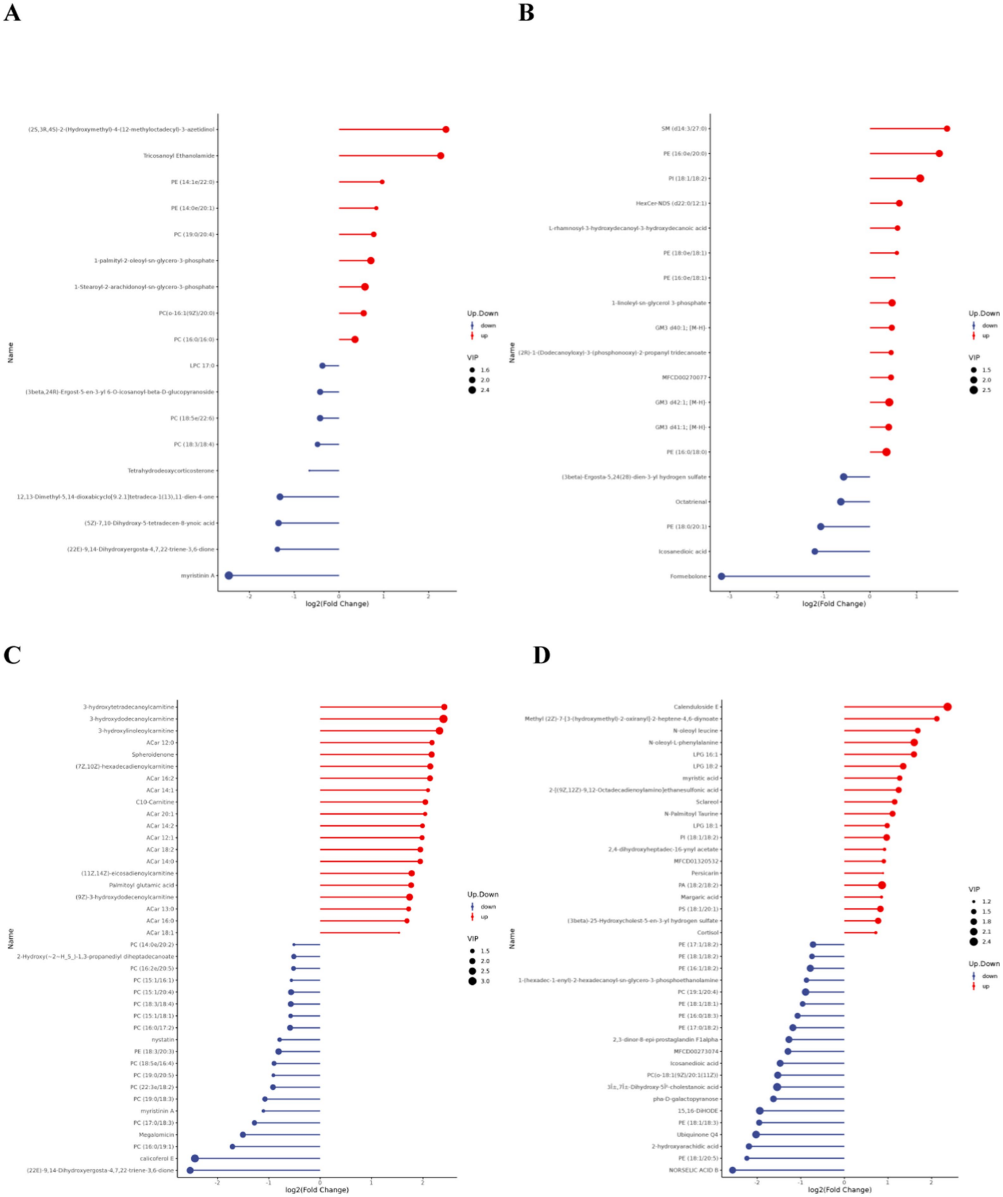
Matchstick diagram of differential lipid metabolites. **(A,C)** are the difference metabolite changes in the pre- and post-competition comparisons between the excellent and normal groups in the positive mode. **(B,D)** are the difference metabolite changes in the pre- and post-competition comparisons between the excellent and normal groups in the negative mode.

Matchstick plots derived from the differential lipid metabolites identified in each comparative group combination provide a clearer representation of the upregulation and downregulation of differential metabolites, as well as substances with significant fold changes. Figure 6 displays the top 20 most upregulated and downregulated differential metabolites.

A total of 18 significantly different lipid metabolites were screened in the pre-game comparison between the excellent group and the normal group in the positive ion mode, of which 9 were up-regulated and 9 were down-regulated (Table 5), and a total of 19 significantly different metabolites were screened in the negative ion mode, of which 14 were up-regulated and 5 were down-regulated (Table 6).

**Table 5:** Pre-competition differential lipid metabolites (in positive ion mode) in the excellent and average groups.

Lipid	RT/min	FC-value	*P*-value	VIP	Lipid classification
FA (23:0)	8.651	+5.2246	0.0293	2.1599	FA
FA (25:0)	10.059	+4.8203	0.0261	2.2227	FA
PE (14:1e/22:0)	14.305	+1.9488	0.0272	1.5216	PE
PE (14:0e/20:1)	13.317	+1.7792	0.0261	1.4158	PE
PC (19:0/20:4)	12.99	+1.7103	0.0434	1.7363	PC
PA (16:0/18:1)	15.498	+1.6358	0.0002	2.3437	PA
PA (18:0/20:4)	13.327	+1.4931	0.0345	2.4578	PA
PC (o-16:1(9Z)/20:0)	13.617	+1.4637	0.0451	1.9544	PC
PC (16:0/16:0)	12.086	+1.2797	0.0267	2.2812	PC
LPC 17:0	3.922	−0.7735	0.0330	1.8815	PC
SL (54:0)	15.774	−0.7462	0.0351	1.8974	SL
PC (18:5e/22:6)	11.89	−0.7458	0.0418	1.9898	PC
PC (18:3/18:4)	12.847	−0.7168	0.0475	1.7281	PC
ST (21:0)	1.666	−0.6325	0.0384	1.2560	ST
C14 H20 O3	1.143	−0.4009	0.0233	2.1360	-
FA (14:2,2OH)	1.145	−0.3922	0.0384	1.9838	FA
ST (28:3,2OH,2 K)	1.698	−0.3855	0.0422	1.7041	ST
myristinin A	7.844	−0.1818	0.0199	2.7392	-

Comparison group data are presented as fold difference (FC) values, with “+” denoting upward and “−” denoting downward adjustments; only the top 20 compounds with upward and downward adjustments are shown in the table, as below.

**Table 6:** Pre-competition differential lipid metabolites (in negative ion mode) in the excellent and average groups.

Lipid	RT/min	FC-value	*P*-value	VIP	Lipid classification
SM (d14:3/27:0)	12.953	+3.1401	0.0496	1.7074	SM
PE (16:0e/20:0)	14.429	+2.7994	0.0146	2.0448	PE
PI (18:1/18:2)	10.507	+2.1076	0.0008	2.3921	PI
HexCer-NDS (d22:0/12:1)	11.139	+1.5456	0.0438	1.8768	HexCer
SL (10:1/10:1)	1.274	+1.5053	0.0486	1.4918	SL
PE (18:0e/18:1)	14.292	+1.4902	0.0467	1.1998	PE
PE (16:0e/18:1)	13.316	+1.4361	0.0313	1.0228	PE
GL (18:2)	2.389	+1.3877	0.0311	2.1055	GL
GM3 d40:1; [M-H]-	13.413	+1.3820	0.0496	1.6787	GM3
GL (12:0/13:0)	11.132	+1.3680	0.0337	1.4045	GL
Sphingosine 1-phosphate	2.301	+1.3660	0.0394	1.6078	SP
GM3 d42:1; [M-H]-	14.282	+1.3313	0.0041	2.4940	GM3
GM3 d41:1; [M-H]-	13.885	+1.3200	0.0393	1.9470	GM3
PE (16:0/18:0)	12.078	+1.2770	0.0108	2.5324	PE
ST (28:2)	7.668	−0.6763	0.0423	2.1572	ST
2,4,6-Octatrienal	1.012	−0.6489	0.0360	2.2058	FA
PE (18:0/20:1)	13.13	−0.4814	0.0053	2.0573	PE
FA (18:0,18:0)	2.944	−0.4409	0.0453	1.8111	FA
Formebolone	1.302	−0.1103	0.0363	2.0952	ST

A total of 75 significantly different lipid metabolites were screened in the post-competition comparison between the excellent group and the normal group in the positive ion mode, of which 52 were up-regulated and 23 were down-regulated (Table 7), and a total of 69 significantly different metabolites were screened in the negative ion mode, of which 36 were up-regulated and 33 were down-regulated (Table 8).

**Table 7:** Differential lipid metabolites (in positive ion mode) in the immediate post-competition period in the excellent and average groups.

Lipid	RT/min	FC-value	*P*-value	VIP	Lipid classification
FA (14:0(OH)/3:0)	1.378	+5.3368	*p* < 0.0001	2.0481	FA
FA (12:0(OH)/3:0)	1.135	+5.2813	*P* < 0.0001	3.0995	FA
FA (18:2 (3-OH)/3:0)	1.653	+5.0057	*P* < 0.0001	2.6945	FA
ACar 12:0	1.297	+4.5176	0.0004	1.7025	ACar
Spheroidenone	6.933	+4.4973	0.0246	2.0131	PK
ACar 16:2(7Z,10Z)/3:0	1.533	+4.4176	0.0005	1.9907	ACar
ACar 16:2	1.536	+4.4040	0.0001	1.9109	ACar
ACar 14:1	1.447	+4.2826	0.0005	1.3939	ACar
C10-Carnitine	1.099	+4.1337	0.0002	1.8631	ACar
ACar 20:1	4.81	+4.1220	0.0070	1.4234	ACar
ACar 14:2	1.253	+3.9790	0.0010	1.5869	ACar
ACar 12:1	1.179	+3.9559	0.0007	1.6378	ACar
ACar 18:2	2.108	+3.8606	0.0004	1.8761	ACar
ACar 14:0	1.74	+3.8558	0.0016	1.8012	ACar
ACar 20:2(11Z,14Z)/3:0	3.257	+3.4355	0.0018	2.0660	ACar
FA (16:0-Glu)	1.231	+3.4126	0.0002	1.8767	FA
FA (12:1(9Z)-OH/3:0)	1.071	+3.3406	0.0004	2.4114	FA
ACar 13:0	1.405	+3.2973	0.0049	1.6239	ACar
ACar 16:0	2.681	+3.2269	0.0105	1.6527	ACar
ACar 18:1	2.937	+2.8990	0.0076	1.1938	ACar
PC (14:0e/20:2)	13.611	−0.7016	0.0495	1.5780	PC
GL 12:0/12:0 (2-OH)	0.919	−0.7005	0.0136	1.2911	GL
PC (16:2e/20:5)	12.808	−0.6975	0.0089	1.9551	PC
PC (15:1/16:1)	11.536	−0.6776	0.0290	1.9270	PC
PC (15:1/20:4)	11.426	−0.6749	0.0279	1.5209	PC
PC (18:3/18:4)	12.847	−0.6726	0.0296	1.9572	PC
PC (15:1/18:1)	12.812	−0.6711	0.0230	1.5047	PC
PC (16:0/17:2)	11.42	−0.6661	0.0382	2.1890	PC
nystatin	13.65	−0.5784	0.0162	1.6142	-
PE (18:3/20:3)	11.599	−0.5703	0.0041	1.3372	PE
PC (18:5e/16:4)	11.543	−0.5364	0.0336	1.8589	PC
PC (19:0/20:5)	12.216	−0.5313	0.0230	1.7142	PC
PC (22:3e/18:2)	13.677	−0.5283	0.0363	1.3254	PC
PC (19:0/18:3)	12.371	−0.4740	0.0021	1.7108	PC
myristinin A	7.844	−0.4647	0.0161	1.9964	-
PC (17:0/18:3)	11.175	−0.4115	0.0339	1.7883	PC
Megalomicin	11.712	−0.3521	0.0199	2.9061	-
PC (16:0/19:1)	13.562	−0.3061	0.0192	2.4200	PC

**Table 8:** Differential post-competition lipid metabolites (in negative ion mode) between the excellent and average groups.

Lipid	RT/min	FC-value	*P*-value	VIP	Lipid classification
Calenduloside E	1.374	+5.1601	0.0012	2.4063	SL
FA (24:1)	6.913	+3.2112	0.0396	1.5516	FA
LPG 16:1	1.903	+3.0233	0.0136	1.5931	PG
LPG 18:2	2.119	+2.5478	0.0045	2.1124	PG
FA (14:0)	4.482	+2.4097	0.0149	1.7211	FA
Sclareol	7.381	+2.2243	0.0498	1.8273	-
N-Palmitoyl Taurine	2.491	+2.1515	0.0048	1.5235	FA
LPG 18:1	2.95	+1.9753	0.0264	1.6857	PG
PI (18:1/18:2)	10.507	+1.9587	0.0168	1.5709	PI
oleoylglycine	4.315	+1.8727	0.0251	1.6615	FA
Persicarin	0.999	+1.8491	0.0482	1.4984	-
PA (18:2/18:2)	10.381	+1.8199	0.0029	1.8697	PA
Margaric acid	7.692	+1.8054	0.0477	1.2286	FA
PS (18:1/20:1)	12.673	+1.7737	0.0370	1.3487	PS
ST (27:0)	1.813	+1.7113	0.0168	1.1724	ST
Cortisol	1.057	+1.6488	0.0226	2.3069	ST
PE (17:1/18:2)	11.009	−0.6078	0.0045	2.0113	PE
PE (18:1/18:2)	11.606	−0.5980	0.0095	1.5202	PE
PE (16:1/18:2)	10.42	−0.5823	0.0151	2.1030	PE
PE (16:1/16:0)	0.935	−0.5478	0.0410	1.5786	PE
PC (19:1/20:4)	11.696	−0.5402	0.0259	1.6737	PC
PE (18:1/18:1)	12.564	−0.5155	0.0215	1.9695	PE
PE (16:0/18:3)	10.746	−0.4755	0.0063	1.9497	PE
PE (17:0/18:2)	12.174	−0.4414	0.0014	1.9024	PE
GL (18:1)	1.788	−0.4082	0.0228	1.9407	GL
FA (20:0,20:0)	2.944	−0.3606	0.0133	2.0026	FA
PC (o-18:1(9Z)/20:1(11Z))	13.285	−0.3470	0.0420	2.3356	PC
pha-D-galactopyranose	1.288	−0.3240	0.0123	1.8484	SL
15,16-diHOME	1.313	−0.2610	0.0179	2.2011	FA
PE (18:1/18:3)	10.821	−0.2584	0.0271	1.6943	PE
Ubiquinone Q4	1.807	−0.2458	0.0048	2.1775	-
2-hydroxyarachidic acid	3.379	−0.2195	0.0090	1.7427	FA
PE (18:1/20:5)	10.677	−0.2125	0.0386	1.4630	PE
Norselic acid B	1.661	−0.1693	0.0209	1.9333	FA

**Figure 7:**
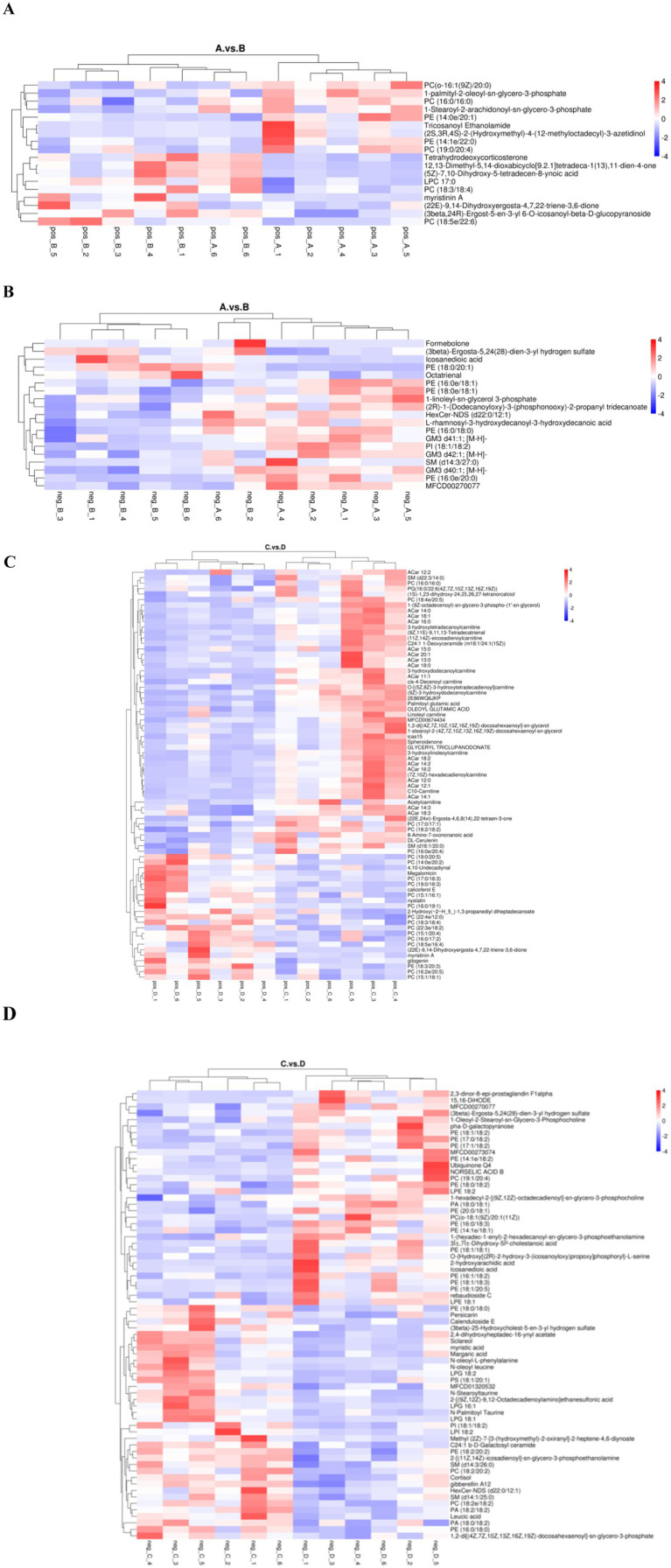
Cluster analysis plot of differential lipid metabolites. **(A,C)** are pre- and post-game cluster analyses of the excellent and average groups in the positive mode. **(B,D)** are pre- and post-game cluster analyses of the excellent and average groups in the negative mode.

Hierarchical clustering analysis of differential lipid metabolites was performed to derive the differences in metabolic expression patterns between and within two groups for the same comparative pair, vertically for samples and horizontally for compounds, with shorter cluster branches representing higher similarity. As shown in Figure 7, the samples in each group can be well clustered together, and the metabolites with similar variations are clustered in the same cluster, indicating that the hierarchical clustering of metabolites with relative quantitative values between the groups is distinctly differentiated, and that the screening of the different metabolites has a certain degree of reliability.

**Figure 8:**
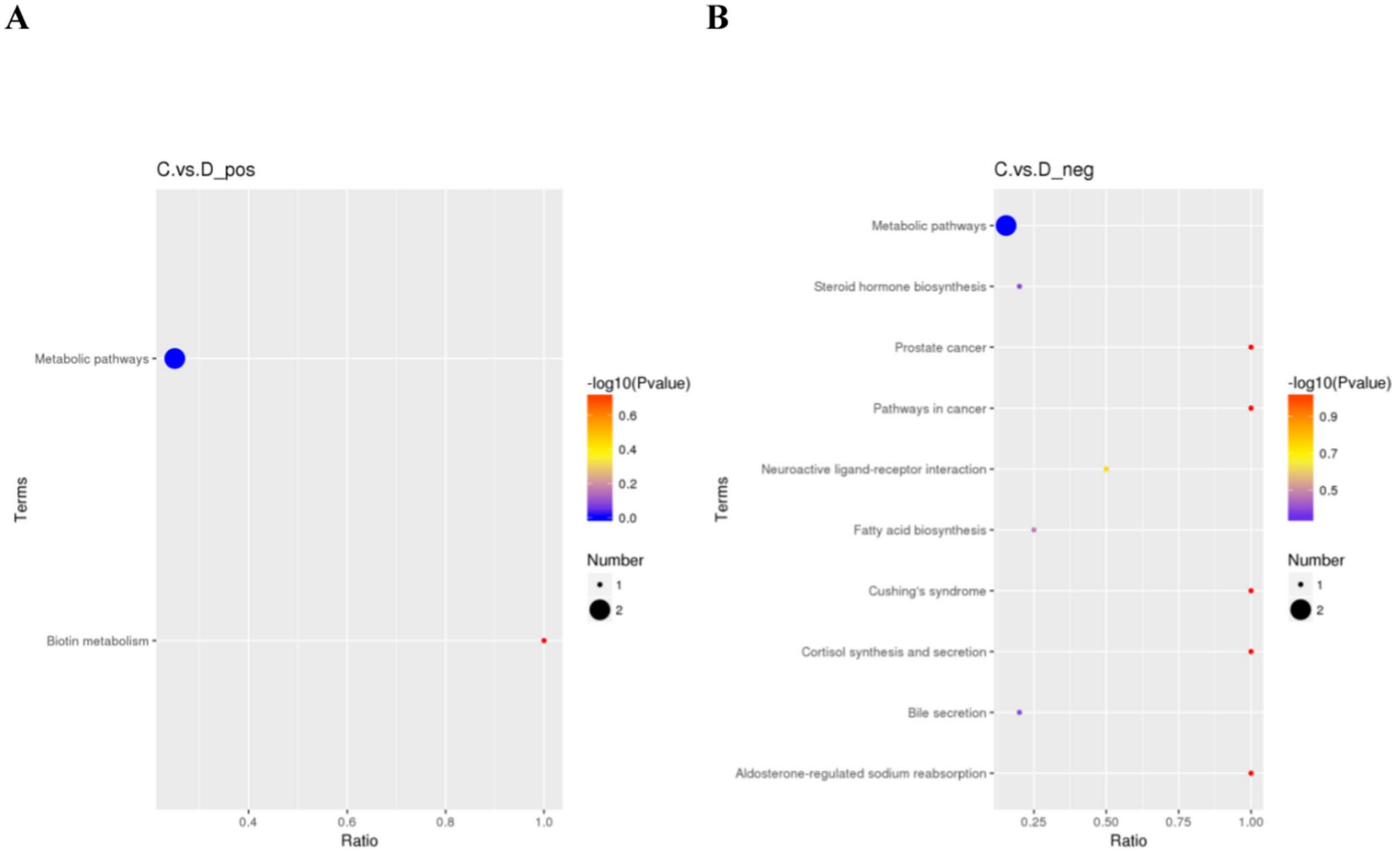
Bubble diagram of KEGG enrichment analysis. **(A)** is the bubble plot of immediate post-game KEGG enrichment analysis for the excellent and average groups in the positive mode; **(B)** is the bubble plot of immediate post-game KEGG enrichment analysis for the excellent and average groups in the negative mode.

With the help of hypergeometric test, the *p*-value of pathway enrichment was obtained, where *p*-value ≤ 0.05 was used as the threshold, and KEGG pathways meeting this condition were defined as KEGG pathways significantly enriched in differential metabolites and plotted in a bubble diagram (Figure 8). KEGG pathway enrichment analysis can identify the main biological functions exercised by differential metabolites, and a total of seven exercise-related pathways were screened in this study. Differential metabolites were mainly enriched in the post-competition period, and the above metabolites were significantly enriched in the biotin metabolic pathway in the positive ion mode, and in six pathways, namely, steroid hormone biosynthesis, neuroactive ligand-receptor interactions, fatty acid biosynthesis, cortisol synthesis and secretion, bile secretion, and aldosterone-regulated sodium reabsorption, in the negative ion mode.

#### 3.3.3 Correlation analysis of blood biochemical indices with differential lipid metabolites

**Figure 9:**
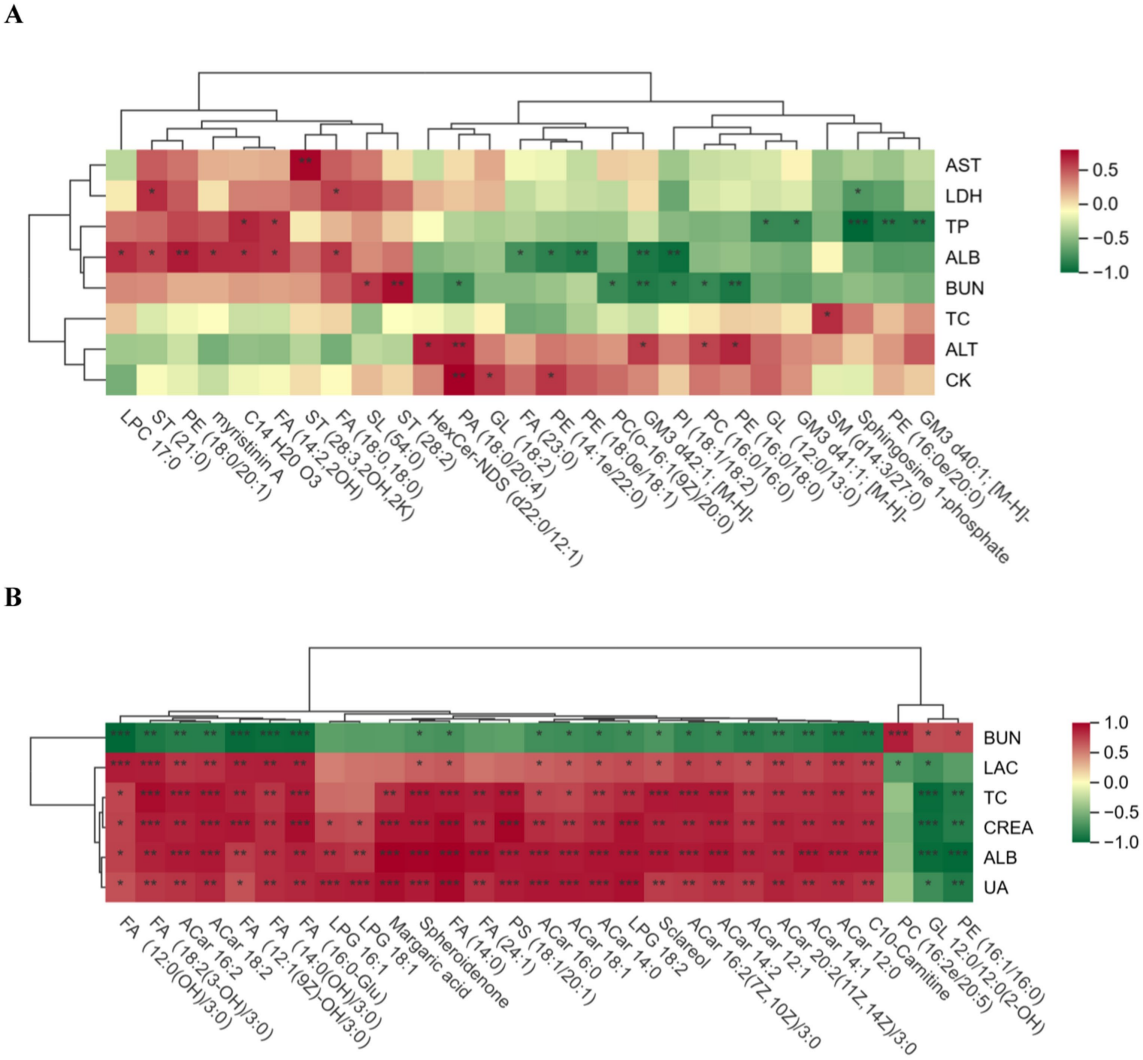
Correlation plot of biochemical indicators and differential lipid compounds. **(A)** is the pre-competition correlation graph between the excellent and average groups, and **(B)** is the post-competition correlation graph between the excellent and average groups. **(A)** “*” in the box in the graph indicates significant, and “**” and “***” indicate highly significant.

The biochemical indices and differential lipid metabolites of the horses in the excellent group vs. the ordinary group before and after the race were correlated, and the correlation between the groups was analysed by calculating pearson correlation coefficients between the biochemical indices and the differential lipid metabolites. The significance level *p* < 0.05 was chosen as the threshold for significant correlation. When the linear relationship between the two groups is strengthened, the positive correlation tends to be 1 and the negative correlation tends to be −1. Red indicates positive correlation and green indicates negative correlation, and the darker the colour, the closer the correlation is to 1. It can be seen in Figure 9 that the correlation between biochemical indices and differentiated lipid metabolites was relatively weak in the pre-race period, but the correlation was very significantly strengthened and most of them were positively correlated in the post-race period.

## 4 Discussion

### 4.1 Differential lipid metabolite analysis of glycerophospholipid (GP) species

GP are major components of biological membranes (23). Phosphatidic acid (PA) is the simplest cytosolic glycerophospholipid and is not abundant in eukaryotic cells (24), which is consistent with the results of the present study in which only nine PAs were found. In blood biochemistry, ALT was significantly higher in the excellent group before the race than in the ordinary group, and it also remained high in the immediate aftermath of the race. There is a relevant literature that shows that ALT is mainly concentrated in the hepatocytes, heart and skeletal muscle. The level of ALT is one of the markers that responds to athletic fatigue, and the significant elevation of ALT is associated with prolonged exercise (25). Normally, lipid metabolism will be activated during and after endurance exercise to provide the energy to cope with the metabolic and physiological related changes in the body produced by prolonged exercise (26), PA is one of the main lipids involved in energy supply, and the content of PA (18:2/18:2) in the excellent group was extremely significantly higher than that of the ordinary group after exercise, which proved that the excellent group possessed a better recovery ability than the ordinary group, and could obtain a greater supply of energy from PA, as well as recovering more quickly after a race to restore the integrity of the cell membrane and the Functionality. In the correlation analysis of pre-competition blood biochemistry and differential lipid metabolites, pre-competition PA (18:0/20:4) showed a significant positive correlation with ALT and CK, and these phenomena may indicate that, compared with the ordinary group, the excellent group, due to long-term systematic training, has skeletal muscle fibres that are more tolerant to high-intensity exercise, which reduces exercise-induced micro-muscle injuries, whereas the muscles of the ordinary group have poorer adaptation and are more prone to excessive release of exercise intensity from ALT, which is also explained by the increase in CK in horses in the excellent group compared to the ordinary group after endurance exercise (27). The values of LDH, CK and AST in the ordinary group before and after exercise were higher than those in the excellent group, but there was no statistical difference, the reason may be that for endurance horses, high-intensity aerobic exercise increases the blood biochemical parameters related to LDH, CK and AST in order to maintain the balance of blood supply to the myocardium (28), and that the values of LDH, CK and AST can be used to diagnose the degree of muscle damage (29), since the horses in the average group have lower physical functioning than the horses in the excellent group, the values of LDH, CK and AST are relatively higher.

Other classes of glycerophospholipids are glycerophospholipids formed by esterification of a phosphate group with several alcohols (choline, ethanolamine, serine, inositol or glycerol): phosphatidylcholine (PC), phosphatidylethanolamine (PE), phosphatidylserine (PS), phosphatidylinositol (PI) and phosphatidylglycerol (PG) (30). In a study by Anna et al. (31), it was found that the levels of lipid metabolites such as PC and PE were significantly reduced in marathon runners after an endurance event, and in the present study, the differential metabolites after the race were found to be significantly lower than the levels of 13 PCs and 6 PEs in the excellent group, and highly significant in the levels of 2 PCs and 4 PEs in the ordinary group. Both before and after the tournament it was found that PCs (18:3/18:4) were significantly downgraded in the excellent group compared to the ordinary group, which may be attributed to the fact that phosphatidylcholine, which contains unsaturated fatty acid chains, is more readily hydrolysed by phospholipase A2 (PLA2) to generate free fatty acids and lysophospholipids at rest (32), which in endurance exercise accelerates fatty acid synthesis used to participate in the subsequent fatty acid oxidation for energy supply. As the main structural components of erythrocyte membranes, fatty acids affect the fluidity and toughness of cell membranes (33, 34). The excellent group had better body quality, more fluid cell membranes, and relatively faster rates of PC synthesis and decomposition compared with the ordinary group, so that the content of PCs (18:3/18:4) was up-regulated in the post-event period compared with that in the pre-event period. Correlation analyses in the excellent and normal groups after the race also found that one of the differential lipid metabolites, PE (16:1/16:0), was significantly negatively correlated with ALB, which proved that there was a link between the increase in ALB levels and the decrease in lipid metabolites, such as PE, after endurance exercise, and it was hypothesised that the decrease in the levels of PC and PE in the excellent group after the race was probably due to the fact that horses in the excellent group were more rapid in the rate of lipolysis, and the ability to use fatty acid oxidation to produce energy was higher than that of the excellent group, so that PC levels were upwardly adjusted to that before the race. The ability to use fatty acid oxidation to produce energy is higher than that of the average group. It is noteworthy that studies for PC (18:3/18:4) have only been reported in potato and hepatocellular carcinoma cells (35–37), and few have been reported in horses. As potential biomarkers of endurance performance in Yili horses that we identified, the mean pre-race PC (18:3/18:4) concentration in the excellent group in the present study was 19964513.22 nmol/mL, and the mean post-race concentration was 19607265.4 nmol/mL; the mean pre-race concentration in the ordinary group was 27850938.95 nmol/mL, and the mean post-race concentration was 29152678.27 nmol/mL.

ALB was significantly higher in the ordinary group than in the excellent group before the endurance event, but was significantly higher in the excellent group than in the ordinary group after the event, and the reason for this result may be the difference in the physical quality of the horses. The main energy-supplying substance for prolonged endurance exercise is fat, and due to the poor water solubility of fatty acids, they are mainly transported in the blood with albumin as the carrier, and the body mobilises fatty acids in large quantities during exercise, resulting in an increase of albumin in the plasma (38). In lipid metabolomics, the level of PI (18:1/18:2) was significantly higher in the excellent group before and after the race than in the ordinary group race, but the FC value was 0.1323 higher before and after the race. PI is a precursor of second messenger molecules and can be hydrolysed to produce the second messenger molecules diacylglycerol (DAG), inositol-1,4,5-trisphosphate and arachidonic acid, which are bioactive molecules precursors (39), so both the excellent and the normal groups provide energy to the body by hydrolysis of PI to produce DAG and other substances during the race, but the excellent group has a greater ability to hydrolyse PI compared to the normal group. PI (18:1/18:2) was also used as a candidate biomarker, which has also been reported only in chicken studies (40), and our results showed that the mean pre-race and post-race concentrations of PI (18:1/18:2) in the excellent group were 64300201.46 nmol/mL, and 65856564.96 nmol/mL, respectively; and the mean pre-race and post-race concentrations of PC in the ordinary group were 30509304.84 nmol/mL, 33623235.9 nmol/mL. Meanwhile, through the correlation analysis of pre-race biochemistry and lipidomics, PI (18:1/18:2) was significantly negatively correlated with ALB, indicating that the increase of ALB after the race was accompanied by the decrease of PI level, and the difference of ALB between the excellent group and the ordinary group showed that the body fat of the excellent group provided more fatty acids than that of the ordinary group, which required more albumin as a carrier for transport.

### 4.2 Differential analysis of lipid metabolites of fatty acid (FA) species

It has been shown that FA play a crucial role in the athlete’s organism, and aerobic exercise increases FA mobilisation in adipose tissue and lipid transport between organs and tissues, improves insulin sensitivity and mitochondrial optimisation, and promotes FA oxidation as an energy substrate (33, 34). FA synthesis is regulated by the enzyme acetyl-coenzyme A carboxylase (ACC), and oxidation of FA produces acetyl-coenzyme A NADH, and FADH2, which are oxidatively phosphorylated to produce ATP (41). In this study, six FAs, including FA (20:0,20:0) and FA (14:0), were highly significant higher than those of the general group and four FAs were significantly higher than those of the general group after the race in the excellent group, which may indicate that the fatty acid mobilisation as well as lipid transport capacity of the horses in the excellent group was significantly higher than that of the ordinary group, and that the oxidation of fatty acids was fully utilised to produce energy for the organism’s energy supply in endurance exercise. CREA is a product of muscle metabolism, and is closely related to the CREA is a product of muscle metabolism, which is closely related to the total amount of muscle in the body (42, 43). In the correlation analysis of biochemical indexes and differential lipid compounds after the race, six differential lipid metabolites, including FA (18:2(3-OH)/3:0), FA (12:1(9z)-OH/3:0), FA (16:0-Glu), and FA (14:0), were positively correlated with CREA, which suggests that the horses are more capable of mobilising fatty acids during endurance exercise than the ordinary group. Lipids stored in the myocytes of horses during endurance exercise are also involved in oxidative energy supply (44), while horses in the excellent group have more muscle mass and relatively higher creatinine production rate and fatty acid oxidative capacity than those in the ordinary group. Meanwhile, the present study found that the level of 15,16-diHOME decreased significantly after the race. 15,16-diHOME is a lipofactor which contributes to the enhancement of FA oxidation for early recovery of skeletal muscle (45), and its regulatory mechanism remains to be further investigated.

Acylcarnitines (ACar) are FAs bound to carnitine, an amino acid derivative that allows FAs transported to mitochondria to be oxidised and contributes to cellular energy conversion. It has been demonstrated that acute endurance exercise causes an increase in acylcarnitines (21), which is consistent with the results of the present study where no statistically significant differences in acylcarnitine content were detected in the pre-race comparison, while a highly significant increase in 14 ACar, including ACar 16:2, ACar 14:1, and C10-Carnitine, was observed in the post-race. The highly significant difference in ACar levels between the two groups of horses after endurance events is likely to be related to the accelerated glycogen depletion and increased reliance on lipolysis for ATP production during endurance events (12). Increased lipolysis leads to higher FA concentrations and subsequently to higher ACar concentrations in the mitochondria, and horses in the ordinary group had lower fat mobilisation and oxidised lipids due to the longer duration of the race and faster glycogen depletion leading to highly significant differences in ACar levels with the excellent group. After high intensity exercise, the increase in ALB content contributes to the retention of fluid in the circulating fluid to maintain homeostasis (46), while ALB was highly significantly and positively correlated with 10 ACar including ACar16:2, ACar18:2, and ACar16:0 after the race, and these findings further suggest the need to pay attention to the oxidative capacity of equine athletes with respect to lipid oxidation for the purpose of better performance in endurance races.

### 4.3 Differential analysis of lipid metabolites of sphingolipid (SP) species

Alterations in SP metabolism and its metabolites lead to mitochondrial dysfunction, autophagy damage, *β*-amyloid dysregulation, and disruption of neuronal homeostasis, which further leads to an increase in reactive oxygen species (ROS) production (47). Sphingomyelin (SM) is the most abundant SP in cells and is ubiquitously distributed in mammalian tissues; SM is an essential element of the plasma membrane and its level is critical for cellular function. In the present study, a statistically significant difference in SM (d14:3/27:0) was detected between the two groups of horses before the endurance event, but no statistically significant difference in SM was detected after the event, which may be attributed to the fact that high-intensity endurance exercise depletes most of the SM used to participate in cellular repair. SM can be converted to ceramide (Cer) by neutral sphingomyelinase (NSM) and acidic sphingomyelinase (ASM). Cer is the simplest SM, a second messenger for lipids, and a precursor of several complex sphingolipids that regulate cell growth and apoptosis (48). No significant difference in Cer was detected between pre-race and post-race in the two comparisons, which may indicate that there is no significant difference in Cer consumption between horses of different exercise levels in a 26 km endurance race.

Gangliosides (GM) are sphingolipid-containing salivary acids that are polymorphic in structure and function and are widely distributed in the human body (49). Three differentially significant GM3 were detected in the excellent group compared to the ordinary group before the race, and not detected after the race, the reason may be that acute endurance exercise caused GM to regulate the body’s immune function resulting in a decrease in GM levels, and the differentially differentiated lipid compounds GM3d42:1 were also found in the correlation analysis of biochemical indexes and plasma differentially differentiated lipid compounds before the race; the significant negative correlation between [M-H]- and ALB, and it is also it can be further suggested that horses in the excellent group possessed better organismal immune regulation compared to horses in the ordinary group, which may have an effect on the performance in endurance races.

### 4.4 Analysis of lipid metabolism signalling pathways

Both long-distance and short-distance competitions cause the horse to develop a stress response during exercise, and cortisol, a hormone closely related to stress, plays a crucial role in exercise (50). Elevated levels of cortisol produce a range of physiological effects that help the body to adapt to prolonged stimulation, but at the same time excess cortisol disrupts the balance between anabolic and catabolic hormones and increases fatigue in exercising horses (51). The reason why the different metabolites in the synthesis and secretion of cortisol signalling pathway were enriched in the excellent group and the ordinary group after the race may be that horses with different sport performance, under prolonged and high-intensity exercise, all need to adapt to the stimulation of the organism by endurance racing through the secretion of cortisol, but it is possible that the ordinary group is not as good as the excellent group at regulating the stimulation of the organism, and so compared with the excellent group, the ordinary group needs to synthesise more cortisol to adapt to the to the stimulation caused by the endurance race.

Following exercise tolerance, differential lipid metabolites are enriched in the pathway, aldosterone, which regulates the reabsorption of Na^+^. Aldosterone is a steroid hormone that regulates renal reabsorption of Na^+^ and therefore plays an important role in the maintenance of water-salt balance (52). Prolonged exercise expels a large amount of sweat resulting in dehydration of the body, which increases fatigue (53) and in severe cases results in the inability of the horse to run the full distance. In this endurance race, the average time to complete the race in the ordinary group was higher than that of the excellent group by 1,133 s, indicating that the ordinary group relied more on aldosterone regulation than the excellent group to maintain the water-salt balance of the body during the race.

Fatty acid biosynthesis is a pathway for the production of fatty acids and involves a variety of enzymes (54). In the analysis of fatty acid metabolites in the present study, it was found that the dependence of horses on fatty acid oxidation as an energy substrate for organismal function increased considerably during long-distance exercise, obtaining energy through the fatty acid biosynthesis pathway.

Alonso et al. (55) on bile acid metabolism mentioned that exercise has a large effect on bile acid metabolism. In the present study, the significant enrichment of differential lipid metabolites in the metabolic pathway of bile secretion may be explained by the fact that endurance exercise led to a decrease in the serum concentration of total bile acids, which in turn caused bile secretion.

## 5 Conclusion

In this study, we focused on the differences in lipid metabolic profiles and biochemical compositions of blood before and after endurance races in Yili horses with different exercise performance through the application of lipid metabolomics and biochemical indicators. A total of 1,537 differential lipid metabolites were screened out, which were relatively concentrated in glycerophospholipids, fatty acids and sphingolipids. These differential lipid metabolites were mainly enriched in metabolic and signalling pathways such as fatty acid biosynthesis, cortisol synthesis and secretion, bile secretion, aldosterone regulation of sodium reabsorption, biotin metabolism, steroid hormone biosynthesis, and neuroactive ligand-receptor interactions. Meanwhile, PC (18:3/18:4) and PI (18:1/18:2) were screened as potential biomarkers to identify the endurance exercise performance of Yili horses, but their specific regulatory mechanisms need to be further investigated. These findings could contribute to the improvement of equine endurance performance and endurance breed selection.

## Data Availability

The original contributions presented in the study are included in the article/supplementary material, further inquiries can be directed to the corresponding author.

## Footnotes

^1^ https://cloud.metware.cn/#/home
